# The relationship between job crafting and job burnout among primary and secondary school physical education teachers: the chain mediating roles of positive psychological capital and work engagement

**DOI:** 10.3389/fpsyg.2026.1889722

**Published:** 2026-08-04

**Authors:** Min Zeng, XiaoFeng Hu

**Affiliations:** 1 Physical Education College, Southwest Minzu University, Chengdu, Sichuan, China; 2 School of Training, Chengdu Sport University, Chengdu, Sichuan, China

**Keywords:** job burnout, job crafting, physical education teachers, positive psychological capital, work engagement

## Abstract

**Objective:**

This study tested a chain mediation model examining the concurrent associations between job crafting and job burnout among primary and secondary school physical education teachers, with a focus on the sequential mediating roles of positive psychological capital and work engagement.

**Methods:**

A cross-sectional design was used. Participants were primary and secondary school physical education teachers in China, recruited through stratified sampling. Standardized scales measured job crafting, positive psychological capital, work engagement, and job burnout. After controlling for demographic variables such as age and gender, the bootstrap method was applied to examine the chain mediating pathways.

**Results:**

Job crafting showed a significant negative association with job burnout (*β* = −0.384, *p* < 0.001). Mediation analysis indicated that job crafting was indirectly associated with burnout through three pathways: through the mediating role of positive psychological capital (*β* = −0.128, *p* < 0.001); through the mediating role of work engagement (*β* = −0.042, *p* < 0.001); and through the sequential mediating pathway of positive psychological capital and work engagement (*β* = −0.044, *p* < 0.001). The total indirect coefficient was −0.214, which accounted for 55.72% of the total association. Bootstrap confidence intervals for all indirect pathways excluded zero, indicating that these mediating pathways were statistically significant.

**Conclusion:**

This study found a negative association between job crafting and job burnout among primary and secondary school PE teachers, with positive psychological capital and work engagement serving as significant mediators both independently and in sequence. Job crafting was associated with lower burnout through higher psychological capital, through higher work engagement, and through a chain pathway from psychological capital to work engagement. These findings integrate the Conservation of Resources theory and the Job Demands-Resources model, outlining a possible psychological process from behavioral adjustment to resource accumulation and motivational activation. The chain mediation model provides a new theoretical perspective for understanding the internal associations between job crafting and burnout, and demonstrates the applicability of this integrated framework in an Eastern cultural context.

## 1 Introduction

Globally, the teaching profession is facing increasing work-related stress and mental health challenges (Hofmann et al., 2022). Physical education (PE) teachers, as key implementers of school health education, are often burdened with multiple demanding tasks, including teaching, training, organizing sports events, and student management (Liu et al., 2026). As a result, job burnout is a particularly salient issue in this group. Job burnout not only shows associations with teachers’ physical and mental health, but also with declines in teaching quality, strained teacher–student relationships, and even higher turnover intentions (Gaudreault et al., 2024). For these reasons, exploring the factors related to burnout and potential mechanisms underlying these associations among primary and secondary school PE teachers has become an important topic in educational psychology and health psychology (Choi et al., 2025).

Among the many factors linked to job burnout, job crafting, a bottom-up proactive behavior strategy, has drawn considerable attention in recent years (Tims et al., 2013). Job crafting refers to the process by which individuals actively change the meaning and boundaries of their work by adjusting tasks, relationships, and cognitive appraisals, thereby improving person–job fit (Tims et al., 2013). Research has shown that job crafting is associated with higher levels of career adaptability and job satisfaction (Kooij et al., 2017). However, whether and through which pathways job crafting is related to burnout, particularly in the specific population of PE teachers, has not been fully explored. It is worth noting that positive psychological capital and work engagement may play important mediating roles in this relationship. Clarifying whether a sequential mediating pathway exists among these variables is essential for building a more complete theoretical model.

From a theoretical perspective, job crafting may help individuals accumulate psychological resources such as hope, resilience, self-efficacy, and optimism, which are the core components of positive psychological capital (Cenciotti et al., 2017). According to the Conservation of Resources theory, the more resources individuals acquire at work, the better they are able to cope with stress and prevent resource depletion, which in turn is related to a lower risk of burnout (Hobfoll et al., 2018). At the same time, higher positive psychological capital may be linked to higher levels of work engagement, characterized by greater vigor, dedication, and absorption in one’s work (Paek et al., 2015). Work engagement is not only an indicator of a positive psychological state but is also considered a key buffer against burnout (Bakker et al., 2014). Therefore, job crafting may be indirectly associated with lower burnout through a sequential pathway of “accumulating psychological capital → increasing work engagement.”

Based on the above reasoning, this study aims to test a chain mediation model: that is, whether job crafting is associated with job burnout through the sequential mediating roles of positive psychological capital and work engagement among primary and secondary school PE teachers. If supported by the data, this model would provide an integrated behavioral–psychological resource framework for understanding the mechanisms related to teacher burnout. At the practical level, the model suggests a possible multi-stage intervention pathway: encouraging teachers to engage in job crafting may be linked to higher levels of psychological capital and work engagement, and through these, to lower burnout. This offers preliminary theoretical support for designing occupational health promotion programs for PE teachers.

## 2 Literature review and hypothesis development

### 2.1 Job crafting and job burnout

Job burnout refers to a psychological syndrome characterized by emotional exhaustion, depersonalization, and a reduced sense of personal accomplishment that arises from prolonged exposure to work-related stressors (Maslach and Leiter, 2016). Among primary and secondary school physical education (PE) teachers, job burnout is particularly prominent due to multiple factors, including highly repetitive teaching tasks, high pressure from student management, insufficient sports resources, and relatively low social recognition (Sarros and Sarros, 1992).

Job crafting refers to the behavioral strategy by which individuals proactively adjust their work tasks, work relationships, and cognitive perceptions of their work in order to achieve a better person–job fit (Wrzesniewski and Dutton, 2001). According to the Job Demands-Resources (JD-R) model, job crafting is theorized to be associated with gaining more job resources (e.g., autonomy, social support) and reduce job demands, which in turn is associated with lower burnout (Bakker and Demerouti, 2017). Even under high workload, active adaptation to the work environment and the reshaping of work roles show positive associations with perceived occupational well-being (Mushtaq and Mehmood, 2023). The core mechanism is that job crafting is conceptually linked to teachers’ adjustment to their work roles according to their own preferences and actual abilities, which is related to lower emotional exhaustion and diminished feelings of reduced personal accomplishment among PE teachers (Dreer, 2022). In addition, the role of job crafting is reflected in an enhanced sense of professional fulfillment and higher work engagement (Mushtaq and Mehmood, 2023). Therefore, Hypothesis 1 (H1) is proposed: Job crafting shows a significant negative association with job burnout among PE teachers.

### 2.2 Job crafting, positive psychological capital, and job burnout

As an important personal resource, psychological capital encompasses four dimensions—self-efficacy, hope, optimism, and resilience, and is considered to buffer the negative association between job demands and burnout, as well as to be positively linked to work engagement (Luthans and Youssef-Morgan, 2017). Existing research has shown that job crafting is theorized to be associated with increased structural resources and challenging demands while decreasing hindrance demands, and this pattern has been shown to be associated with reduced burnout (Tims et al., 2012). Furthermore, job crafting behaviors have been found to be positively related to employees’ levels of psychological capital. For example, proactively seeking resources and challenges has been found to be positively associated with employees’ self-efficacy and resilience (Alessandri et al., 2018). When employees successfully acquire new job resources or cope with challenges through job crafting, they experience a sense of mastery, which is associated with the self-efficacy belief of “I am capable” (Van den Heuvel et al., 2015). Employees with higher psychological capital are more likely to adopt active coping strategies when facing high job demands, which is related to lower emotional exhaustion and depersonalization (Galanakis and Tsitouri, 2022). Vogt et al. (2016) found that employees’ job crafting behaviors were associated with increases in psychological capital 1 year later, and these increases were further associated with lower burnout levels 2 years later. In summary, psychological capital represents a core pathway in the association between job crafting and lower job burnout: job crafting is linked to the accumulation of personal psychological capital, which in turn is indirectly related to a weaker association between job demands and burnout. Therefore, Hypothesis 2 (H2) is proposed: Positive psychological capital mediates the association between job crafting and job burnout.

### 2.3 Job crafting, work engagement, and job burnout

Job crafting, as a behavior through which individuals proactively adjust their work characteristics, is theorized to be associated with increased structural resources (e.g., autonomy, feedback) and challenging demands, while decreasing hindrance demands (Bakker et al., 2012). This behavior is theorized to be associated with the motivational pathway in the JD-R model, whereby job resources, by satisfying individuals’ basic psychological needs, are associated with work engagement, a positive work-related state characterized by vigor, dedication, and absorption (Schaufeli and Bakker, 2004). Specifically, gaining more autonomy or social support through job crafting shows positive associations with experiences of competence and belonging, which are concurrently associated with a greater willingness to fully engage in their work. Actively seeking challenging tasks (e.g., taking on new projects) is positively associated with the sense of meaningfulness at work, which is also linked to higher work engagement (Schaufeli et al., 2002).

According to the Conservation of Resources (COR) theory, individuals are motivated to acquire, retain, and protect their resources (Hobfoll, 2011b). Work engagement, as a positive psychological state, can itself serve as an important resource-gain mechanism. Higher work engagement is positively associated with energy, positive emotions, and a sense of accomplishment, potentially forming a resource gain spiral that is associated with buffers against resource loss (Hakanen et al., 2006). In contrast, low work engagement is associated with higher levels of emotional exhaustion and depersonalization, which are core symptoms of job burnout. Relevant research has confirmed a negative association between work engagement and job burnout (Schaufeli et al., 2002; Zhang et al., 2021). A study among female teachers also showed that work engagement fully mediated the sequential relationship between professional identity and burnout (Sun et al., 2022). Integrating the JD-R model and COR theory, the mediating pathway of ‘job crafting → work engagement → job burnout’ can be explained as follows: job crafting, by optimizing work characteristics, is theorized to be associated with higher work engagement; and the resource accumulation and positive emotions that accompany high work engagement may, in turn, relate to reduced energy depletion along the health impairment path, which is a mechanism theoretically linked to lower burnout. Therefore, Hypothesis 3 (H3) is proposed: Work engagement mediates the association between job crafting and job burnout.

### 2.4 Job crafting, positive psychological capital, work engagement, and job burnout

The previous sections discussed how job crafting is associated with job burnout through psychological capital and work engagement, respectively. However, in real work contexts, individuals’ internal psychological states and behavioral motivations do not operate in isolation; rather, they are jointly associated with job burnout. Although previous research has separately confirmed a positive link from psychological capital to work engagement (Mao and Xie, 2013), and a negative link from work engagement to job burnout (Mushtaq and Mehmood, 2023), studies that examine psychological capital and work engagement as a sequential mediating chain are still relatively scarce.

From an integrated perspective of COR theory and the JD-R model, a more dynamic view is that job crafting may be positively associated with psychological capital; this enhanced psychological capital may, in turn, be positively associated with work engagement; and work engagement, through its motivational function, may be associated with lower burnout. Specifically, through mastery experiences and meaning reconstruction, job crafting is theoretically expected to be positively associated with employees’ self-efficacy, hope, optimism, and resilience—i.e., psychological capital (Sesen and Ertan, 2020). According to COR theory, individuals with abundant psychological resources tend to approach their work in a more positive and engaged manner, because their resource reserves provide the confidence and energy to cope with challenges (Hobfoll, 2011a). Employees with high psychological capital believe they are capable of performing their jobs, hold positive expectations for the future, can design multiple pathways to achieve their goals, and recover quickly from setbacks. These states are considered a core psychological foundation associated with work engagement. (vigor, dedication, absorption) (Kotzé, 2018). Empirical research has shown significant positive associations between psychological capital and work engagement in teacher populations, even when temporal factors are considered (Prieto et al., 2008). Furthermore, highly engaged individuals continuously experience a sense of accomplishment and positive emotions, which is associated with reducing the energy depletion process along the health impairment path and is associated with significantly lower risks of emotional exhaustion and depersonalization (Bakker et al., 2014). Thus, work engagement serves as a key intermediate link between psychological capital and job burnout.

**Figure 1:**
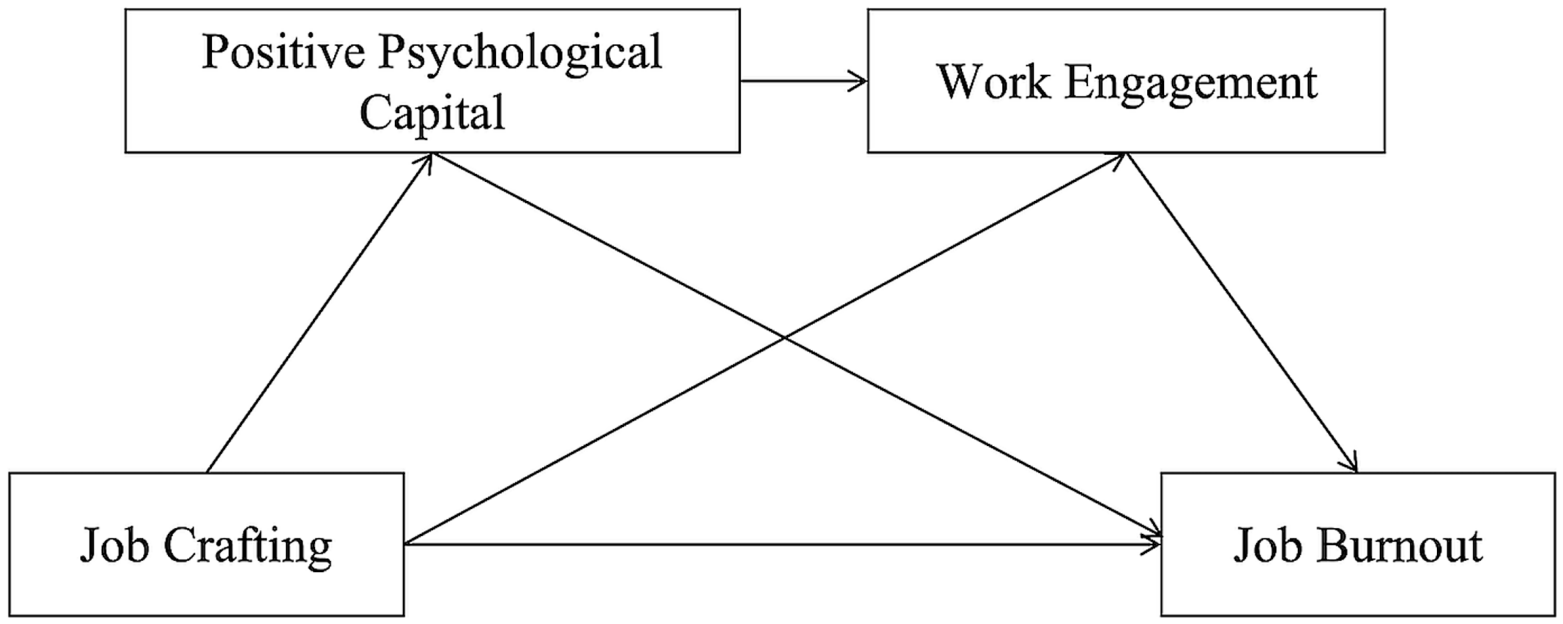
The chain mediation effect model.

The core purpose of testing this “job crafting → psychological capital → work engagement → job burnout” chain mediation model is to examine whether the theoretical pathway from behavioral adjustment to resource accumulation to motivational activation and ultimately to lower burnout is supported. The theoretical basis for this sequential relationship lies in the dynamic transformation of psychological resources: job crafting is theoretically linked to the accumulation of psychological capital; psychological capital, as a personal resource, provides the internal energy source for work engagement; and work engagement, as a motivational state, represents a more proximal mechanism that shows a direct association with burnout. If this pattern of associations is supported by the data, it would point to potentially key sequential steps—from behavioral intervention (promoting job crafting) to resource building (enhancing psychological capital) to motivational strengthening (increasing work engagement)—for future longitudinal studies or intervention programs aimed at exploring causal mechanisms. Therefore, Hypothesis 4 (H4) is proposed: Positive psychological capital and work engagement play a chain mediating role in the association between job crafting and job burnout among primary and secondary school PE teachers (see Figure 1. The chain mediation effect model).

## 3 Methodology

### 3.1 Procedures and participants

This study used a cross-sectional design. From March 1 to March 31, 2026, a total of 1,100 adult physical education (PE) teachers from primary and secondary schools were recruited through stratified sampling in four provinces and municipalities in Southwest China: Sichuan, Yunnan, Guizhou, and Chongqing. The questionnaires were distributed by individuals such as heads of PE departments. Prior to distribution, the research team provided specialized training covering key points of the questionnaire design, including voluntary participation, the withdrawal mechanism, and confidentiality requirements.

Subsequently, the heads of PE departments or other responsible persons distributed the questionnaires to the PE teachers. Participants completed the questionnaires under the supervision of these responsible persons, who explained the voluntary nature of participation, the content of the items, and the right to withdraw. After data collection, 90 invalid responses were excluded based on pre-established criteria: (1) abnormally short or long completion times; (2) inattentive response patterns (e.g., repetitive or contradictory answers).

The final sample consisted of 1,010 participants, yielding a valid response rate of 91.82%. Participants’ ages ranged from 21 to 59 years (M = 35.5, SD = 8.25). The sample included 615 males (60.89%) and 395 females (39.11%). The age distribution was as follows: 21–29 years, 388 participants (38.42%); 30–39 years, 282 participants (27.92%); 40–49 years, 245 participants (24.26%); and 50–59 years, 95 participants (9.40%). Data were collected via an online survey platform^1^. The electronic questionnaire consisted of five sections: basic information (age, gender, years of work experience, marital status, professional title, etc.), a job crafting scale, a positive psychological capital scale, a work engagement scale, and a job burnout scale. Before data collection, all participants provided electronic informed consent. After data collection, all information was anonymized to ensure privacy. The anonymized dataset will be securely stored according to institutional ethical guidelines and data management policies, and will be available for scientific research purposes for at least 5 years after publication. Qualified researchers may apply for access to the data, subject to signing a formal data sharing agreement that ensures the privacy of the participants and compliance with the original informed consent terms.

### 3.2 Ethics approval and consent to participate

This study was conducted in strict accordance with the ethical principles of the Declaration of Helsinki. The research protocol was approved by the Ethics Committee of Southwest Minzu University (approval number: SMU-202601107). Before formal participation, all participants provided electronic informed consent through the online platform. The informed consent form detailed the research purpose, the voluntary nature of participation, the confidentiality agreement, and the right to withdraw from the study at any time without any liability. To protect participant privacy, data were anonymized during collection and analysis, and no personally identifiable information was retained in the final dataset.

### 3.3 Measurement tools

#### 3.3.1 Job crafting scale

Job crafting was measured using the Job Crafting Scale developed by Leana et al. (2009). This scale is unidimensional and consists of 4 items (e.g., “Introduce new approaches to improve my work”; “Change minor work procedures that I think are not productive”). Items are rated on a 5-point Likert scale (1 = strongly disagree, 5 = strongly agree), The total score is calculated by summing the scores of all four items, ranging from 4 to 20. Higher total scores indicate a higher level of perceived job crafting among individuals. With higher scores indicating greater congruence between the item description and one’s own experience. In this study, the scale demonstrated a Cronbach’s alpha of 0.782, χ^2^/df = 1.747, CFI = 0.999, AGFI = 0.991, RFI = 0.990, IFI = 0.999, RMSEA = 0.027. We selected this scale for its focus on task-level behavioral adjustments, which directly capture the types of proactive changes that PE teachers in school settings are realistically able to make.

#### 3.3.2 Positive psychological capital scale

Positive psychological capital was measured using the Psychological Capital Questionnaire (PCQ) developed by Luthans et al. (2004), with the Chinese version revised by Kuo et al. (2010). The scale consists of four dimensions, namely self-efficacy, hope, resilience, and optimism, with a total of 26 items. For scoring, the total or average score of items within each dimension is first calculated to reflect respondents’ level on that specific dimension. Afterwards, the scores of all 26 items are summed to generate an overall scale score. The total score ranges from 26 to 182, and higher scores correspond to higher levels of individuals’ positive psychological capital. Self-efficacy (7 items, e.g., “I feel confident in representing my work area in meetings”), hope (6 items, e.g., “I can think of many ways to reach my current work goals”), resilience (7 items, e.g., “I usually take stressful things at work in stride”), and optimism (6 items, e.g., “I always look on the bright side of things”). Items are rated on a 7-point Likert scale (1 = strongly disagree, 7 = strongly agree), with higher scores indicating greater congruence with the respondent’s own experience. In this study, the scale demonstrated a Cronbach’s alpha of 0.812, χ^2^/df = 1.159, CFI = 0.997, AGFI = 0.970, RFI = 0.973, IFI = 0.997, RMSEA = 0.013.

#### 3.3.3 Work engagement scale

Work engagement was measured using the Utrecht Work Engagement Scale (UWES) developed by Schaufeli et al. (2002). This scale consists of three dimensions (vigor, dedication, absorption) with a total of nine items. The overall scale score is computed by summing the scores of all nine items. The total score ranges from 9 to 54, and higher scores indicate higher levels of individual work engagement. Vigor (3 items, e.g., “At my work, I feel bursting with energy”), dedication (3 items, e.g., “I am enthusiastic about my job”), and absorption (3 items, e.g., “I get carried away when I am working”). Items are rated on a 6-point Likert scale (1 = never, 6 = always), with higher scores indicating that the item description better matches the respondent’s own experience. In this study, the scale demonstrated a Cronbach’s alpha of 0.857, χ^2^/df = 1.356, CFI = 0.996, AGFI = 0.993, RFI = 0.979, IFI = 0.996, RMSEA = 0.019.

#### 3.3.4 Job burnout scale

Job burnout was measured using the Maslach Burnout Inventory (MBI) developed by Maslach (2003). This scale consists of three dimensions: emotional exhaustion, cynicism, and reduced personal accomplishment, with a total of 15 items. The subscale of reduced personal accomplishment uses reverse scoring. Prior to calculating the total score, all reverse-scored items are recoded. The overall score is obtained by summing the recoded scores of all 15 items. The total score ranges from 0 to 90, and higher scores indicate more severe job burnout. Emotional Exhaustion (5 items, e.g., “I feel emotionally drained from my work”), Cynicism (4 items, e.g., “I have become less interested in my work since I started this job”), and Reduced Personal Accomplishment (6 items, e.g., “I can effectively solve the problems that arise in my work”). Items are rated on a 6-point scale (0 = never, 6 = every day), with higher scores indicating greater congruence between the item description and the respondent’s own experience. In this study, the scale demonstrated a Cronbach’s alpha of 0.920, χ^2^/df = 1.602, CFI = 0.993, AGFI = 0.975, RFI = 0.976, IFI = 0.993, RMSEA = 0.024.

### 3.4 Data analysis

The data analysis procedure consisted of three steps.

First, common method bias assessment: Following recent methodological recommendations that Harman’s single-factor test has limited sensitivity in detecting common method bias, this study employed the Unmeasured Latent Method Construct (ULMC) technique as the primary assessment. Specifically, a baseline measurement model was specified with all items loading onto their respective theoretical constructs; a common method factor was then added to this model, with all items loading onto both their substantive construct and the method factor, and the method factor’s variance was fixed to 1 and specified as orthogonal to all trait factors. Model fit comparisons between the baseline and ULMC models were examined to determine whether common method bias posed a serious threat. For reference, Harman’s single-factor test was also conducted as supplementary evidence.

Second, descriptive statistics and correlation analysis: Means, standard deviations, and Pearson correlation coefficients were calculated for all variables (job crafting, positive psychological capital, work engagement, and job burnout).

Third, chain mediation analysis: The PROCESS macro (version 4.0) in SPSS 25.0 was used to construct a chain mediation model. This model examined the indirect pathways linking job crafting to job burnout through the mediating effects of positive psychological capital and work engagement. Prior to the mediation analysis, we conducted a series of hierarchical regression analyses to examine the individual associations of job crafting, positive psychological capital, and work engagement with job burnout. In each regression model, the independent variable (job crafting, positive psychological capital, or work engagement) was entered in a single block using the ‘Enter’ method, while controlling for demographic variables including gender and age. The bootstrap method with 5,000 resamples and 95% confidence intervals was applied to examine the indirect associations.

## 4 Results

### 4.1 Common method bias test

Because the questionnaire data were obtained through self-report at a single time point, common method bias (CMB) is a potential concern. Following the comprehensive framework recommended by Podsakoff et al. (2003) and recent methodological suggestions that Harman’s single-factor test lacks sensitivity in detecting CMB, this study employed the Unmeasured Latent Method Construct (ULMC) technique as the primary assessment. ULMC was implemented following established procedures. First, a baseline measurement model was specified with all items loading onto their respective theoretical constructs (job crafting, positive psychological capital, work engagement, and job burnout). Second, a common method factor was added to the model, with all items loading onto both their theoretical construct and this unmeasured latent method factor. The method factor’s variance was fixed to 1, and it was specified as orthogonal to all trait factors.

As shown in Table 1, the baseline model showed acceptable fit, while the ULMC model demonstrated slightly improved fit. According to the established criteria, significant CMB is indicated when ∆CFI ≥ 0.01 or ∆RMSEA ≥ 0.015. In this study, both ∆CFI = 0.007 and ∆RMSEA = 0.001 fell well below these thresholds. These results confirm that common method bias does not pose a serious threat to the validity of the findings. We also retained the Harman’s single-factor test as supplementary reference, which showed that the first factor explained 28.91% of the total variance (below the 40% threshold), further supporting this conclusion.

**Table 1:** Comparison of model fit indices between baseline model and ULMC model.

Model	χ^2^/df	CFI	TLI	RMSEA	∆CFI	∆RMSEA
Baseline model (without method factor)	2.710	0.908	0.902	0.041		
ULMC model (with method factor)	2.612	0.915	0.907	0.040	0.007	0.001

∆CFI = CFI_ULMC−CFI_Baseline; ∆RMSEA = RMSEA_Baseline−RMSEA_ULMC.

### 4.2 Descriptive statistics and correlations

Descriptive statistics and Pearson correlation coefficients were calculated for the four variables (job crafting, positive psychological capital, work engagement, and job burnout). Significant intercorrelations were found among all variables (see Table 2).

**Table 2:** Descriptive statistics and correlation analysis of variables.

Variable	*M*	SD	Cronbach’s alpha	Job crafting	Positive psychological capital	Work engagement	Job burnout
Job crafting	11.99	2.270	0.782	1			
Positive psychological capital	103.96	17.866	0.812	0.558**	1		
Work engagement	26.97	5.854	0.857	0.369**	0.438**	1	
Job burnout	44.97	10.753	0.920	−0.384**	−0.426**	−0.397**	1

**p < 0.01.

Job crafting was negatively correlated with job burnout (*r* = −0.384, *p* < 0.01). Job crafting was positively correlated with positive psychological capital (*r* = 0.558, *p* < 0.01) and with work engagement (*r* = 0.369, *p* < 0.01). Positive psychological capital was positively correlated with work engagement (r = 0.438, p < 0.01) and negatively correlated with job burnout (*r* = −0.426, *p* < 0.01). Work engagement was negatively correlated with job burnout (*r* = −0.397, *p* < 0.01).

### 4.3 Job crafting and job burnout among PE teacher: chain-mediated effects

To further examine the mediating effects, we used Model 6 of the PROCESS macro with 5,000 bootstrap resamples to test the chain mediation model. In this model, job burnout was specified as the dependent variable, job crafting as the independent variable, and positive psychological capital and work engagement as the parallel and sequential mediators, while controlling for gender and age.

The regression results are presented in Table 3. Job crafting significantly and positively predicted positive psychological capital (*β* = 4.373, *p* < 0.001) and work engagement (*β* = 0.467, *p* < 0.001), and significantly and negatively predicted job burnout (*β* = −0.806, *p* < 0.001). Positive psychological capital significantly and positively predicted work engagement (*β* = 0.111, *p* < 0.001) and significantly and negatively predicted job burnout (*β* = −0.138, *p* < 0.001). Work engagement significantly and negatively predicted job burnout (*β* = −0.431, *p* < 0.001).

**Table 3:** Regression analysis of job crafting, positive psychological capital, work engagement, and job burnout.

Outcome variable	Predictor variable	*β*	*t*	*R*	*R*2	*F*
Positive psychological capital				0.560	0.313	153.068
	Gender	1.656	1.772			
	Age	0.019	0.450			
	Job crafting	4.373	21.238***			
Work engagement				0.464	0.215	68.917
	Gender	−0.336	−1.024			
	Age	−0.009	−0.586			
	Job crafting	0.467	5.376***			
	Positive psychological capital	0.111	10.060***			
Job burnout				0.506	0.226	69.055
	Gender	−0.025	−0.043			
	Age	0.012	0.455			
	Job crafting	−0.806	−5.114***			
	Positive psychological capital	−0.138	−6.637***			
	Work engagement	−0.431	−7.630***			

***p < 0.001.

As shown in Table 4, job crafting showed a significant negative association with job burnout among primary and secondary school PE teachers (*β* = −0.384, *p* < 0.001; 95% CI [−0.327, −0.041]). This indicates that higher job crafting scores were associated with lower job burnout, supporting Hypothesis 1. The association between job crafting and job burnout was mediated by positive psychological capital (indirect coefficient = −0.128, *p* < 0.001; 95% CI [−0.166, −0.088]), indicating that higher job crafting was associated with lower burnout through higher positive psychological capital, thus supporting Hypothesis 2. Work engagement also mediated the association between job crafting and job burnout (indirect coefficient = −0.042, *p* < 0.001), indicating that job crafting was associated with lower burnout through higher work engagement, which supports Hypothesis 3. Finally, positive psychological capital and work engagement played a sequential mediating role in this association (indirect coefficient = −0.044, *p* < 0.001; 95% CI [−0.060, −0.030]), indicating that job crafting was positively associated with positive psychological capital, which in turn was positively associated with work engagement; through this sequential pathway, job crafting also showed an indirect negative association with job burnout. This finding supports Hypothesis 4.

**Table 4:** Mediation effect values and effect sizes.

Effect path	Effect	BOOT SE	BOOT LLCI	BOOT ULCI	Relative mediation effect
Total effect	−0.384***	0.029	−0.441	−0.327	100%
Direct effect	−0.170***	0.033	−0.235	−0.105	44.27%
Total indirect effect	−0.214***	0.022	−0.257	−0.171	55.73%
Indirect effect 1 (positive psychological capital)	−0.128***	0.020	−0.166	−0.088	33.33%
Indirect effect 2 (work engagement)	−0.042***	0.009	−0.062	−0.025	10.94%
Indirect effect 3 (positive psychological capital and work engagement)	−0.044***	0.008	−0.060	−0.030	11.46%

Indirect effect 1: job crafting → positive psychological capital → job burnout; Indirect effect 2: job crafting→ work engagement → job burnout; Indirect effect 3: job crafting → positive psychological capital → work engagement → job burnout. ***p < 0.001.

**Figure 2:**
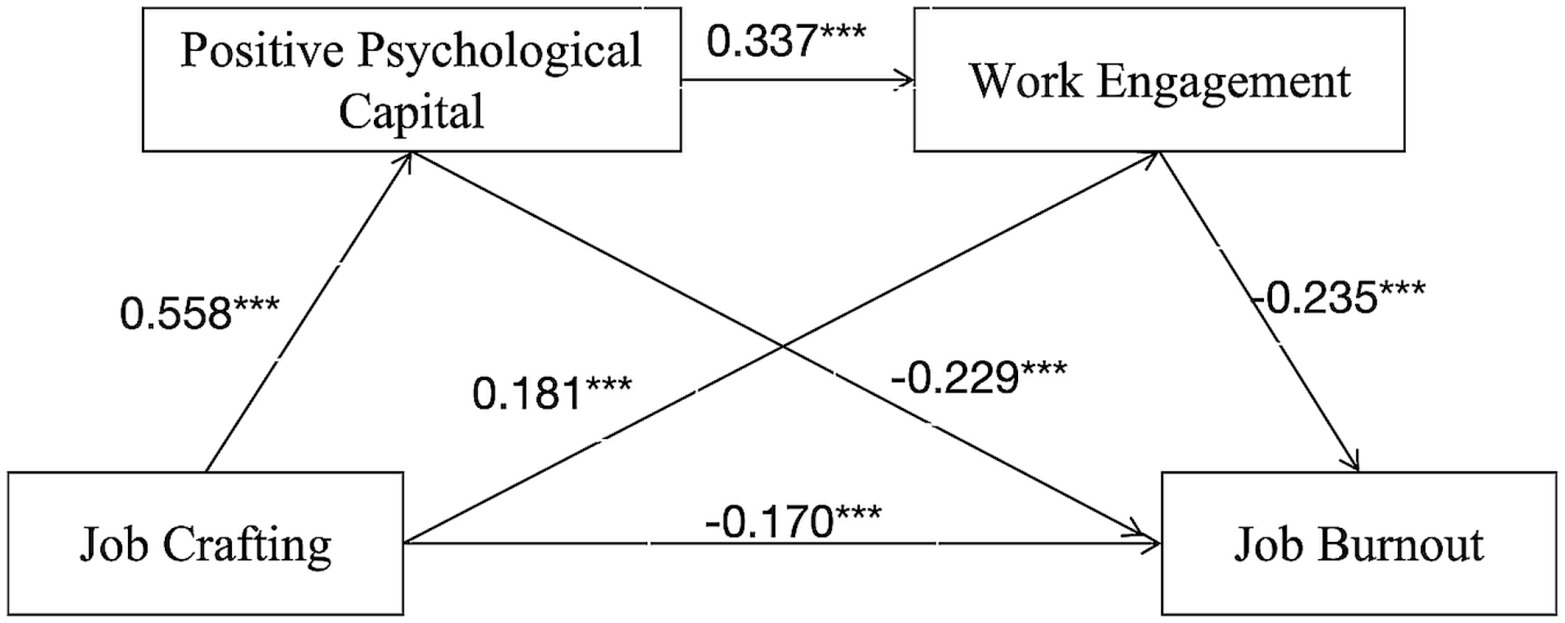
Model of chain mediating roles of positive psychological capital and work engagement between job crafting and job burnout.

All three indirect pathways and the total effect coefficient were statistically significant (*p* < 0.001). The total association coefficient was −0.384, with a direct coefficient of −0.170 (accounting for 44.27% of the total) and a total indirect coefficient of −0.214 (accounting for 55.73%). Among the indirect pathways: Pathway 1 (job crafting → positive psychological capital → job burnout) had an indirect coefficient of −0.128 (accounting for 33.33%); Pathway 2 (job crafting → work engagement → job burnout) had an indirect coefficient of −0.042 (accounting for 10.94%); and Pathway 3 (job crafting → positive psychological capital → work engagement → job burnout) had an indirect coefficient of −0.044 (accounting for 11.46%). The specific pathways are illustrated in Figure 2.

## 5 Discussion

### 5.1 Main findings

This study examined the association between job crafting and job burnout among primary and secondary school physical education (PE) teachers, and explored the chain mediating roles of positive psychological capital and work engagement within this association. Overall, the data supported the four proposed hypotheses. Job crafting was negatively correlated with job burnout, meaning that teachers with higher levels of job crafting reported lower levels of burnout. Further analyses showed that job crafting was not only directly associated with burnout, but also indirectly related to it through three pathways: the independent mediation of positive psychological capital, the independent mediation of work engagement, and the sequential mediation of both. Although the effect size of this chain mediating pathway was relatively small, such small effect sizes are common in cross-sectional studies and carry clear theoretical meaning. The findings suggest that a sequential pattern, from behavioral adjustment to resource accumulation and then to motivational activation—may co-occur with burnout.

### 5.2 The direct association between job crafting and job burnout

A significant negative correlation was found in this study between job crafting and job burnout among primary and secondary school PE teachers. This finding supports Hypothesis H1 and is consistent with previous research. It also aligns with findings from studies based on the Job Demands-Resources (JD-R) model, which suggest that proactive work adjustment behaviors are often associated with more positive work states and a lower risk of burnout (Bakker and Demerouti, 2007; Demerouti et al., 2001).

Although direct empirical research on PE teachers is relatively limited, recent studies in the broader teaching profession provide support. For example, a systematic literature review clearly identified job crafting as a key behavior through which employees proactively adjust their tasks, environment, and interactions to achieve a better person–job fit. This behavior is considered a strategy associated with better management of job demands, higher job satisfaction and well-being, and lower burnout (Bhopal and Devi, 2026). These findings resonate with the pattern of associations observed in the present sample of PE teachers. However, Most existing studies, including the present one, used a cross-sectional design. Therefore, we can only report a concurrent association between job crafting and burnout and cannot infer that job crafting leads to reduced burnout. Future longitudinal studies or intervention experiments are crucial for clarifying any possible temporal relationships between them.

Furthermore, the present study focused on the specific group of primary and secondary school PE teachers, which adds to the existing literature. PE teachers face unique occupational challenges, such as high physical exertion, repetitive skill instruction, pressure from organizing sports events, and sometimes a marginalized subject status (Shen et al., 2024). This study found that in this context, job crafting behaviors (e.g., introducing new teaching methods or adjusting inefficient work procedures) were still associated with lower reported burnout. This suggests that even in educational environments with relatively limited resources or fixed work patterns, teachers’ proactive behavioral adjustments may be a positively associated factor in their occupational health ecosystem.

From a theoretical perspective, this association provides empirical support for the JD-R model in a specific educational and cultural context (Schaufeli and Bakker, 2004). The model posits that employees are not merely passive recipients of job characteristics, but can also actively shape their work environment to gain resources and reduce unfavorable demands. The present results are consistent with this view, indicating that in the context of primary and secondary PE education in China, teachers’ job crafting behaviors are similarly associated with a more favorable work-related psychological state (i.e., lower burnout). This extends the evidence base for the model to a non-Western, education-specialist domain.

### 5.3 The mediating role of positive psychological capital

The mediation analysis results indicated that positive psychological capital played a significant mediating role between job crafting and job burnout. This finding supports Hypothesis H2 and reveals an important pathway: job crafting behavior was associated with higher levels of positive psychological capital among teachers, and higher psychological capital, in turn, was associated with lower reported burnout. This result is consistent with the Conservation of Resources (COR) theory, which holds that individuals acquire and accumulate psychological resources (e.g., self-efficacy, hope, resilience, and optimism) through proactive behaviors such as job crafting. These resources are thought to be associated with buffering work stress and are thus associated with healthier occupational states (Shi and Rucktum, 2023; Park and Ha, 2025).

Recent cross-sectional evidence in the teaching profession supports this pathway. A cross-sectional study of preschool teachers found that psychological capital played an important mediating role between emotional labor strategies and job burnout, indicating that psychological resources are a key internal factor in the association between work behaviors and burnout (Peng et al., 2019). Although that study focused on emotional labor rather than job crafting, the core mechanism—that individual strategies are associated with burnout through psychological resources, aligns with the pattern found in the present study. Another study with nurses reported a similar pattern: positive psychological capital was highly positively correlated with job crafting, and both were linked to higher work engagement and fewer negative work states (Park and Ha, 2025). These cross-occupational findings together support psychological capital as a core psychological construct linking individual proactive behaviors to occupational health outcomes.

However, it must be emphasized that the majority of existing supporting evidence, including the present study, is based on cross-sectional data. Therefore, this study cannot determine whether job crafting leads to an increase in psychological capital and thereby reduces burnout, or whether teachers with high psychological capital are more likely to engage in job crafting and concurrently experience lower burnout. A plausible interpretation is that these variables may form a dynamic, interconnected ecosystem in teachers’ professional lives.

The present study tested this pathway in the specific context of primary and secondary school PE teachers, which is an important addition to the literature. PE teachers work in environments characterized by high physical demands and multiple task pressures. This study found that even in such environments, teachers’ proactive work adjustment behaviors (job crafting) were still associated with a positive state of psychological capital, and this psychological state coexisted with lower reported burnout. This suggests that higher levels of psychological capital may be a valuable factor associated with the maintenance of occupational health.

### 5.4 The mediating role of work engagement

This study supported the mediating role of work engagement between job crafting and job burnout. The mediation analysis showed an indirect coefficient of −0.042 (accounting for 10.938% of the total association, 95% CI excluding zero) for the pathway through work engagement. This result suggests that work engagement, as a motivational mediator, played a role in the association between behavioral adjustment and burnout.

For instance, Aldrin and Merdiaty found in a study of IT employees that job crafting was not directly associated with work engagement; rather, the association was fully mediated by mindfulness (Aldrin and Merdiaty, 2019). This differs from the partial mediation pattern found in the present study, which may stem from differences in sample characteristics (e.g., occupation, culture) or study design. In addition, a longitudinal study of Chinese medical staff found that mindfulness was associated with increased work engagement, which was associated with subsequent job crafting behavior. This suggests a possible bidirectional relationship between work engagement and job crafting, posing a challenge to the interpretation of work engagement solely as an outcome variable in cross-sectional studies.

Despite these inconsistencies, meta-analytic evidence provides overall support for the mediating pathway of work engagement. Within the framework of the JD-R model, a large body of empirical research has confirmed that job crafting (as a resource-gaining behavior) is primarily associated with work engagement (or disengagement), and that work engagement is negatively correlated with job burnout (Bakker and Demerouti, 2007; Schaufeli and Bakker, 2004). Among teacher populations, Ornaghi et al. also confirmed that work engagement was associated with lower burnout (Ornaghi et al., 2023). A recent study of teachers further found that job crafting was positively associated with teacher well-being through the mediating role of work engagement (Dwijayanthy et al., 2023).

The effect size of work engagement’s mediating role was relatively small. This may be because teachers’ work engagement is more strongly associated with organizational context variables (e.g., school support, difficulty of student management), and the association between individual-level job crafting and work engagement may be limited. Moreover, the cross-sectional design cannot distinguish any bidirectional relationship between work engagement and burnout. Future research could adopt longitudinal designs to further clarify the dynamic relationships among these variables.

### 5.5 The chain mediating role of positive psychological capital and work engagement

A key finding is the support for the chain mediating effect of positive psychological capital and work engagement in the association between job crafting and job burnout. The mediation analysis showed an indirect coefficient of −0.044 (accounting for 11.458% of the total association, 95% CI excluding zero) for the sequential pathway (job crafting → positive psychological capital → work engagement → burnout). This result suggests that an integrated psychological process—from behavioral adjustment (job crafting) to resource accumulation (psychological capital) to motivational activation (work engagement), and finally to its association with lower burnout—is identifiable in theory.

Recent chain mediation studies in teacher populations provide comparable empirical evidence for this sequential pathway. Zhao et al. tested the parallel and chain mediating roles of work engagement and psychological capital between career calling and burnout in a sample of primary and secondary school teachers, finding a significant chain mediating pathway with a relatively small effect size, similar to the pattern observed in the present study (Zhao et al., 2022). Sun et al. also supported the mediating roles of work engagement and psychological capital between teacher professional identity and burnout. Although their model emphasized parallel mediation, it similarly highlighted the importance of these two core variables (Sun et al., 2022). A common feature of these studies is that psychological capital and work engagement exhibited a sequential mediating role between different antecedents and job burnout. This complements the present study’s findings with job crafting as the antecedent. Regardless of whether the starting point for activating teachers’ positive work states is an internal career calling, professional identity, or behavioral job crafting, the sequential pattern of associations of psychological capital and work engagement appears to be part of the mechanism associated with burnout, although the strength of the association varies across studies.

The theoretical plausibility of this sequential pathway can be understood from an integrated perspective of COR theory and the JD-R model. Within the gain spiral framework of COR theory, initial resources (e.g., psychological resources accumulated through job crafting) can facilitate further resource gains, forming an upward gain cycle. As core personal resources, higher levels of psychological capital are associated with a state of work engagement characterized by vigor, dedication, and absorption. Work engagement itself is also an important resource state that can further consolidate and expand one’s resource reservoir, and is thus associated with lower levels of resource loss occurring simultaneously. A longitudinal intervention study provided experimental evidence for the link between personal resources and burnout, showing that positive psychological capital interventions, job crafting interventions, and combined interventions were all associated with lower employee burnout, with the psychological capital and combined interventions showing particularly strong associations with reductions in burnout (Pérez-Marqués et al., 2023). That study offered direct evidence for the practical idea of countering burnout through enhancing personal resources (such as psychological capital) and behavioral adjustments (job crafting). Notably, although the intervention period and follow-up design of that study could not fully separate the dynamic temporal relationship between psychological capital and job crafting in real work settings, the findings provided preliminary experimental support for the possibility that synergistic interventions combining job crafting and psychological capital are more strongly associated with lower burnout.

However, modeling psychological capital and work engagement in a psychological-capital-first, work-engagement-second sequence is not without controversy in the empirical literature. At the micro-level of daily fluctuations, some research suggests that psychological capital and work engagement may have a more synchronous or mutually reinforcing relationship. Dudasova et al., in a two-wave longitudinal study of teachers, found that perceived social support was associated with higher psychological capital 2 years later, and that increases in psychological capital were positively associated with changes in work engagement, supporting a temporal sequence in which psychological capital precedes work engagement (Dudasova et al., 2024). On the other hand, Uslukaya and Zincirli explicitly identified a reciprocal, cross-lagged relationship between job resources and work engagement in their longitudinal study of teachers (Uslukaya and Zincirli, 2025). These findings suggest that the directional specification from psychological capital to work engagement in the chain mediation model tested in this study, although consistent with the resource-to-engagement gain spiral logic of COR theory, may in actual temporal dynamics be closer to a mutually reinforcing coupling relationship. Because this study used a cross-sectional design, the concurrent associations among variables cannot reveal the temporal order between psychological capital and work engagement. The directional hypothesis awaits examination in longitudinal research.

### 5.6 Theoretical contributions

By constructing and testing a chain mediation model of positive psychological capital and work engagement between job crafting and job burnout, this study makes theoretical contributions on several levels.

First, this study extends the perspective of job crafting research to the specific occupational group of primary and secondary school PE teachers. Previous research has mostly focused on general occupational groups or corporate employees, with insufficient attention paid to PE teachers working in high-intensity, multi-task environments. This study found support for the negative association between job crafting and burnout in this group, offering a new perspective for understanding the mechanisms related to the formation and alleviation of burnout among PE teachers.

Second, this study integrates COR theory and the JD-R model to construct a complete theoretical model—from behavioral adjustment to resource accumulation to motivational activation—that is associated with job burnout. Previous research has often explained the job crafting–burnout relationship from a single theoretical perspective, and seldom integrated multiple theories within one framework. Through theoretical integration, this study revealed multiple pathways in the association between job crafting and burnout: a resource pathway through positive psychological capital, a motivational pathway through work engagement, and a sequential transmission pathway through both.

Third, this study revealed the sequential associative pattern between positive psychological capital and work engagement, responding to the lack of attention in existing research to the linked relationships among mediators. Previous research has often examined the independent role of a single mediator, and less often explored the theoretically sequential relationships among multiple mediators. Building on the resource gain spiral mechanism of COR theory, this study proposed and found support for a sequential mediating pathway of positive psychological capital and work engagement. This finding deepens the understanding of the mechanisms linking job crafting and burnout, suggesting that positive psychological capital is not only a mediator between job crafting and burnout, but is also indirectly associated with burnout through work engagement.

Fourth, this study extends the cultural context. Previous research on the job crafting–burnout relationship has mostly been conducted in Western cultural contexts. Using a sample of Chinese primary and secondary school PE teachers, this study provided evidence for the applicability of the relevant theoretical model in an Eastern cultural context. This cross-cultural verification helps examine the universality of the theory and provides a basis for future cross-cultural comparative research.

### 5.7 Limitations and future directions

This study has several limitations that need to be considered when interpreting the findings and that point to directions for future research.

First, there are limitations related to the research design. This study used a cross-sectional design, with all variables measured at a single time point; therefore, it is not possible to infer temporal sequences or causal relationships among the variables. Although the sequential pathway of job crafting → positive psychological capital → work engagement → job burnout was proposed based on theoretical reasoning, cross-sectional data cannot confirm this temporal order. Future studies could adopt longitudinal designs, measuring variables at multiple time points, to examine dynamic changes and the directionality of the relationships.

Second, there are limitations related to statistical inference with cross-sectional data. Furthermore, while bootstrapping provides a robust method for testing indirect effects, it does not overcome the inherent limitation of cross-sectional data, which is the inability to establish temporal precedence among variables. Therefore, the confidence intervals reported for the indirect effects should not be interpreted as evidence of causal ordering.

Third, there are limitations related to measurement methods. All variables in this study were measured using self-report questionnaires, which may be subject to common method bias and socially desirable responding. Although control measures such as anonymous completion were implemented, the inherent subjectivity of self-report data may still affect the accuracy of the results. Nevertheless, the ULMC analysis conducted in this study confirmed that common method bias did not pose a serious threat to the validity of the findings (∆CFI = 0.007 < 0.01, ∆RMSEA = 0.001 < 0.015), which mitigates this concern to some extent. Future research could combine multiple measurement methods, such as peer ratings, supervisor ratings, behavioral observations, or ecological momentary assessment, to reduce recall bias.

Fourth, there are limitations related to the sample. The sample was limited to primary and secondary school PE teachers in Southwest China, and caution is needed when generalizing the conclusions to other regions or educational stages. Differences in educational resources and socio-cultural environments across regions may influence the patterns of association among the variables. Future research could expand the sample to other regions, other educational stages (e.g., kindergarten, university), or other occupational groups to examine the applicability of the chain mediation model.

Fifth, there are limitations related to variable control. This study did not include some potential confounding factors that may be associated with the relationships among the variables, such as teaching experience, professional title, school type, weekly teaching hours, and level of social support. Future research should include these variables as covariates in the model to more accurately estimate the net associations among the variables. Additionally, the unidimensional job crafting measure used in this study was appropriate for assessing overall task-level behavioral adjustments, but it did not capture the multi-dimensional nature of job crafting as conceptualized in the JD-R framework. Consequently, we could not examine whether specific crafting strategies have differential associations with psychological capital, work engagement, and burnout. Future research employing multi-dimensional measures could provide a more nuanced understanding of these relationships.

Sixth, there are limitations related to the completeness of the model. The chain mediation model examined in this study explained a portion of the mechanism linking job crafting and burnout, but a part of the direct association remained unexplained, suggesting that other mediating pathways may exist. Future research could explore other potential mediators, such as perceived organizational support, emotion regulation strategies, and self-efficacy, to further enrich the understanding of the mechanisms linking job crafting and burnout. It would also be valuable to examine moderating variables, such as gender, age, and years of work experience, to explore whether the model performs differently under different conditions.

Seventh, there are limitations related to cultural factors. This study was conducted among Chinese primary and secondary school PE teachers, and the findings may be influenced by the specific cultural context. The emphasis on collectivism, respect for authority, and face in Chinese culture may give job crafting a unique form and shape its associations with other variables. Future research could conduct cross-cultural comparisons to examine the applicability and similarities or differences of the chain mediation model proposed in this study across different cultural contexts.

## 6 Conclusion

A significant negative association was observed between job crafting and job burnout. Teachers with higher levels of job crafting reported lower levels of burnout. Positive psychological capital played a mediating role in this relationship. Job crafting was associated with lower burnout through positive psychological capital, a finding that aligns with the Conservation of Resources theory. Work engagement also played a mediating role. Job crafting was associated with lower burnout through work engagement, a finding consistent with the Job Demands-Resources model. The results further suggest a chain mediating role of positive psychological capital and work engagement. Job crafting was associated with positive psychological capital, positive psychological capital was associated with work engagement, and work engagement was associated with job burnout. This sequential pathway integrates COR theory and the JD-R model. It outlines a possible psychological process from behavioral adjustment to resource accumulation to motivational activation, and finally to its association with burnout.

The chain mediation model constructed and tested in this study provides a new theoretical perspective for understanding the internal associations between job crafting and job burnout among primary and secondary school PE teachers. The findings indicate that job crafting is associated with burnout not only through a psychological resource pathway but also through the motivational pathway of work engagement. A sequential relationship exists between these two pathways. This finding expands the research perspective on the job crafting–burnout relationship. It shifts the focus from a single mediator to the sequential interplay of multiple variables and offers a reference framework for future research. In addition, this study tested the applicability of the relevant theoretical model in an Eastern cultural context, laying a foundation for cross-cultural comparative research.

## Data Availability

The original contributions presented in the study are included in the article/Supplementary material, further inquiries can be directed to the corresponding author/s.

## Footnotes

1
https://www.wenjuan.com/s/UZBZJvYWmq/#
